# MHCSeqNet2—improved peptide-class I MHC binding prediction for alleles with low data

**DOI:** 10.1093/bioinformatics/btad780

**Published:** 2023-12-28

**Authors:** Patiphan Wongklaew, Sira Sriswasdi, Ekapol Chuangsuwanich

**Affiliations:** Department of Computer Engineering, Faculty of Engineering, Chulalongkorn University, Bangkok 10330, Thailand; Center of Excellence in Computational Molecular Biology, Division of Research Affairs, Faculty of Medicine, Chulalongkorn University, Bangkok 10330, Thailand; Center for Artificial Intelligence in Medicine, Division of Research Affairs, Faculty of Medicine, Chulalongkorn University, Bangkok 10330, Thailand; Department of Computer Engineering, Faculty of Engineering, Chulalongkorn University, Bangkok 10330, Thailand; Center of Excellence in Computational Molecular Biology, Division of Research Affairs, Faculty of Medicine, Chulalongkorn University, Bangkok 10330, Thailand

## Abstract

**Motivation:**

The binding of a peptide antigen to a Class I major histocompatibility complex (MHC) protein is part of a key process that lets the immune system recognize an infected cell or a cancer cell. This mechanism enabled the development of peptide-based vaccines that can activate the patient’s immune response to treat cancers. Hence, the ability of accurately predict peptide-MHC binding is an essential component for prioritizing the best peptides for each patient. However, peptide-MHC binding experimental data for many MHC alleles are still lacking, which limited the accuracy of existing prediction models.

**Results:**

In this study, we presented an improved version of MHCSeqNet that utilized sub-word-level peptide features, a 3D structure embedding for MHC alleles, and an expanded training dataset to achieve better generalizability on MHC alleles with small amounts of data. Visualization of MHC allele embeddings confirms that the model was able to group alleles with similar binding specificity, including those with no peptide ligand in the training dataset. Furthermore, an external evaluation suggests that MHCSeqNet2 can improve the prioritization of T cell epitopes for MHC alleles with small amount of training data.

**Availability and implementation:**

The source code and installation instruction for MHCSeqNet2 are available at https://github.com/cmb-chula/MHCSeqNet2.

## 1 Introduction

Immunotherapy has been around since the late 19th century (McCarthy 2006) and has been one of the focal points in research. One of its primary attractions is its capacity to be engineered for precise targeting of cancer cells or specific cell types in each patient, resulting in fewer side effects compared to chemotherapy and radiotherapy. There are various forms of immunotherapy (Kruger *et al.* 2019), some of which rely on the presentation of foreign peptide antigens via major histocompatibility complex (MHC) molecules on the surface of the cells. This presentation enables the immune system to recognize foreign antigen–MHC complexes and trigger the destruction of the cells.

Among thousands of foreign peptides that are expressed inside a cancer cell or an infected cell, only a handful would be successfully processed through the entire antigen presentation pathway—from proteasome cleavage and MHC binding to cytotoxic T-cell recognition. To date, machine-learning models have been developed to predict the specificities of these steps (Keşmir *et al.* 2002, Phloyphisut *et al.* 2019, O’Donnell *et al.* 2020, Reynisson *et al.* 2020, Wells *et al.* 2020), all of which are important for screening potential immunogenic antigens. Peptide-MHC binding prediction, in particular, has received a lot of interests in the research community due to the abundance of public experimental data (Vita *et al.* 2018).

There are two major classes of MHC proteins: Class I and Class II (Wieczorek *et al.* 2017). Class I MHC proteins play an important role in monitoring peptides generated from proteins inside a cell and displaying them on the cell’s surface. On the other hand, Class II MHC proteins are typically expressed in specific immune-related cells and are associated with presenting both extracellular and endogenous peptides via a phagocytosis or autophagy pathway. While both classes of MHC proteins are relevant in the context of immunotherapy, prediction for Class I MHC binding has been more successful compared to Class II (Chen *et al.* 2019) because Class I antigens are shorter, exhibit more well-defined specificities (Wieczorek *et al.* 2017), and have more training data (Vita *et al.* 2018).

Several types of experimental data can be used to train peptide–MHC binding prediction. Binding affinity and stability measurements, such as dissociation constant, half-maximal inhibitory concentration (IC50), and binding half-life, can inform the model of quantitative binding characteristics. Peptidomics data, which consist of mass spectrometry identifications of peptides extracted from MHC proteins on the cell’s surface, provide a large, qualitative volume of positive antigens. While quantitative binding and affinity data are theoretically more informative, the heterogeneity in experimental techniques and conditions across laboratories can introduce considerable noises. Also, quantitative experimental data were more costly to obtain in bulk. On the other hand, peptidomics data cannot distinguish between weakly- and strongly bound antigens and do not provide negative data.

The architectures of MHC binding prediction models are relatively simple, consisting of a peptide input module, an MHC allele input module, and a prediction head (Andreatta and Nielsen 2015, Xie *et al.* 2019, O’Donnell *et al.* 2020, Reynisson *et al.* 2020). For the input modules, various architectures have been used, such as the recurrent Gated Recurrent Unit (GRU) and Long Short-Term Memory (LSTM) layers in MHCSeqNet (Phloyphisut *et al.* 2019) and MHCherryPan (Xie *et al.* 2019), which can accommodate peptides with variable lengths but are more difficult to train, or the fully connected layers in MHCFlurry (O’Donnell *et al.* 2020) and NetMHCPan (Reynisson *et al.* 2020), which require the input sequences to be aligned and specifically formatted. MHCherryPan also utilized convolutional layers to extract motif features from the input MHC alleles. Once extracted, features from the input peptide and the MHC allele are flattened and concatenated before being sent to the prediction head, which is composed of fully connected layers that output either the binding affinity or the binding probability. Some works utilize an ensemble of these simple architectures to capture the heterogeneity of HLA binding specificities (O’Donnell *et al.* 2020, Reynisson *et al.* 2020) while our approach focuses on building a single network that can generalize.

**Figure 1.**
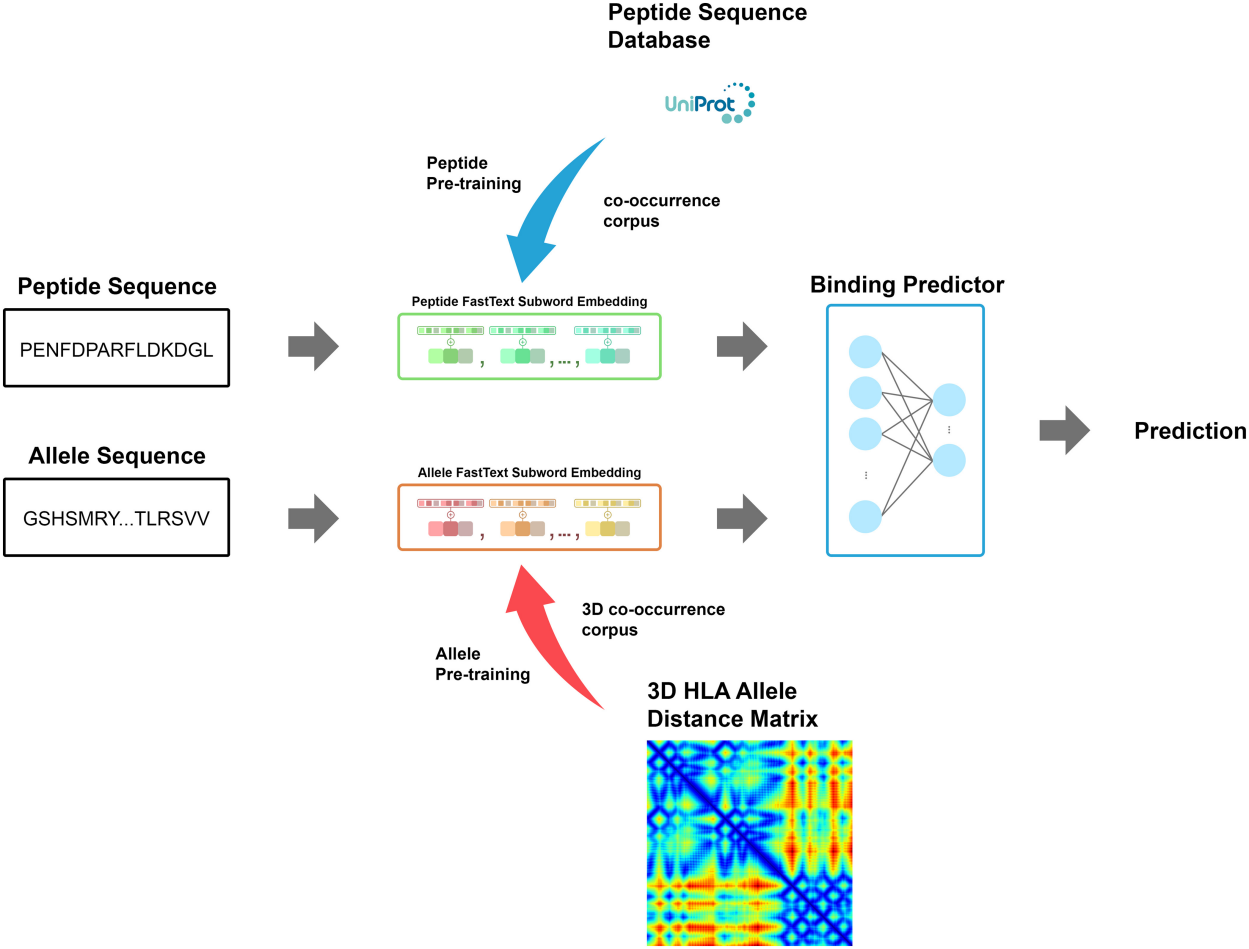
The proposed MHCSeqNet v2 architecture. MHCSeqNet v2 consists of three primary components: the peptide sequence input module, the HLA allele input module, and the binding probability prediction. The two input modules extract features from amino acid sequences using a fastText-styled method, where word representations are formed by aggregating sub-word embeddings. To initialize the input modules, natural peptides and 3D structure information of HLA proteins were used for pre-training. Finally, the embeddings of input peptide and HLA allele are concatenated and fed into a fully connected network for making prediction.

Although several prediction models for peptide-MHC binding have achieved high overall accuracy (O’Donnell *et al.* 2020, Reynisson *et al.* 2020), their performance tends to decrease substantially when evaluated on MHC alleles with limited training data. Our previous work (Phloyphisut *et al.* 2019) diverged from other approaches by focusing on a representation learning strategy for amino acid sequence from the input peptide and MHC allele to allow the model to generalize to MHC alleles with few available training data. Here, sub-word-level feature extraction inspired by fastText (Bojanowski *et al.* 2017) was employed to further improve the generalizability of the model (Fig. 1). The use of sub-words allows unseen or rare amino acid combinations to be encoded as a sum of its more common parts and allows information sharing across related sequences. Furthermore, additional training data obtained from a large-scale re-analysis of public peptidomics datasets (Sricharoensuk *et al.* 2022) was included. Both factors helped MHCSeqNet v2 outperform other approaches on multiple metrics.

## 2 Materials and methods

### 2.1 IEDB HLA binding dataset

The first HLA binding dataset comes from combining several mass spectrometry-based mono-allelic HLA peptidomics studies (Abelin *et al.* 2017, 2019, Marco *et al.* 2017, Solleder *et al.* 2019, Sarkizova *et al.* 2020) with peptide–HLA pairs curated by the Immune Epitope Database (IEDB) (Vita *et al.* 2018). Duplicated peptide–HLA pairs and peptides with modifications were removed. In total, there were 514 928 peptide–HLA pairs across 164 alleles. As mass spectrometry datasets contain only positive data, negative data points were generated as described in Section 2.12.

### 2.2 SMSNet HLA binding dataset

The second HLA binding dataset was obtained from a prior study (Sricharoensuk *et al.* 2022), which used SMSNet (Karunratanakul *et al.* 2019), a *de novo* peptide sequencing tool, to re-analyze two mono-allelic HLA peptidomics datasets (Abelin *et al.* 2017, Sarkizova *et al.* 2020). A total of 43 190 new peptide–HLA pairs across 89 alleles with peptide lengths ranging from 8 to 15 amino acids were identified. The script for combining this dataset with the IEDB dataset is included as part of the source code.

### 2.3 HLA binding affinity dataset

Preprocessed HLA binding affinity data were obtained from MHCFlurry’s curated dataset, which was reportedly aggregated from IEDB (Vita *et al.* 2018) and other data sources. The source code for obtaining the data can be found on MHCFlurry’s GitHub repository (https://github.com/openvax/mhcflurry). The dataset was further cleaned by removing peptides that overlap with an HLA binding dataset and peptides corresponding to non-human MHC proteins. This was done to ensure that our models, which were pre-trained on HLA binding datasets, cannot directly exploit that knowledge to predict affinity scores. Affinity values were transformed to the [0, 1] range using the formula $1-{\;\text{log}\;}_{50000}(x)$ as described in other works (O’Donnell *et al.* 2020, Reynisson *et al.* 2020).

### 2.4 T cell epitope dataset

T cell epitopes were downloaded from IEDB. Entries with modified peptides, peptides containing ambiguous amino acids, or non-specific HLA alleles were removed. Peptide–HLA pairs with conflicting labels (both positive and negative) were also removed. Only entries with peptides ranging from 8 to 15 amino acids in length were kept. This yielded a dataset of 42 534 unique peptide–HLA pairs (33 366 negative and 9168 positive) across 129 alleles. Among these, 182 peptide–HLA pairs correspond to 35 alleles with few HLA binding training data (<200 ligands).

### 2.5 Peptide dataset for pre-training

The collection of natural amino acid sequences for pre-training the peptide embedding module was generated as previously described (Phloyphisut *et al.* 2019). Briefly, proteasome cleavage sites on verified proteins from UniProt (2020) were predicted using NetChop (Keşmir *et al.* 2002). This produced a list of 16 million 9-mer peptides that are flanked by cleavage sites with predicted scores of 0.5 or higher. The permissive threshold of 0.5 (compared to the default of 0.7) was selected to ensure that almost every region of each protein would be sampled. As a result, many overlapping 9-mers were obtained from the same proteins. Hence, the peptide embedding module will be less likely to overfit to particular proteins or particular regions on the proteins.

### 2.6 HLA structure dataset for pre-training

3D structural models of HLA proteins were downloaded from the pHLA3D database (Menezes Teles e Oliveira *et al.* 2019). A pairwise C${}_{\alpha }$–C${}_{\alpha }$ distance matrix was calculated for each allele. Because some portions of the N-terminus and C-terminus were absent in the structural models, only distances between residue positions 24 and 301 were considered. Distance matrices from all HLA alleles were averaged to yield a single context matrix for pre-training the allele embedding module.

### 2.7 HLA allele processing

A multiple sequence alignment of all HLA proteins was constructed using MUSCLE 3.8.31 (Edgar 2004) to map residue positions across alleles. The resulting alignment contains 372 residue positions. The aligned amino acid sequences were then processed as described in Section 2.8.

### 2.8 Words and sub-words extraction from amino acid sequences

Amino acid sequences were processed using a fastText-like architecture (Bojanowski *et al.* 2017). First, the beginning of each sequence was padded with characters  ^ to make the length divisible by the word size (which is three). Words were formed by splitting the amino acid sequences into non-overlapping substrings each with length equals to the word size. For example, an input sequence ABCD would be padded to   ^   ^ ABCD and then split into two words,  ^   ^ A and BCD. Then, a list of sub-words for each word was formed by enumerating all of its substrings. With word size of three, there are six sub-words for each word. For example, the sub-words for BCD would be B, C, D, BC, CD, and BCD itself.

### 2.9 Construction of sequence embedding from words and sub-words

**Figure 2.**
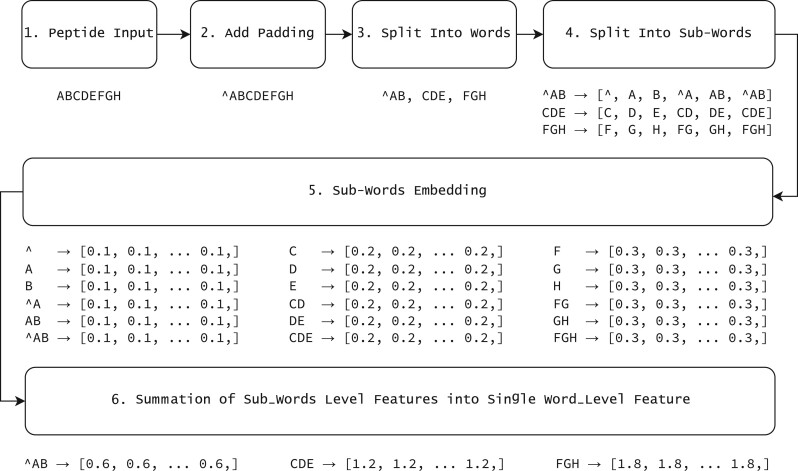
The peptide processing steps used with an example.

Once the embedding for each sub-word is learned, the embedding of each word was calculated by summing the embeddings of all of its sub-words. Finally, the embedding of the original amino acid sequence is defined as the collection of the embeddings of its words. An illustration of this procedure is provided in Fig. 2. For example, the embedding for an input sequence ABCDEFGH would be the collection of the embeddings for the words   ^ AB, CDE, and FGH.

### 2.10 Context words pairing

For each word, its context words were defined in two ways. If the word was extracted from an HLA allele, its context words are those that are located within 45 Å away on the 3D structure of HLA protein. If the word was extracted from a peptide, its context words are those located within three words away on the sequence. For example, given a list of words [  ^   ^A, BCD, EFG, HIJ, KLM] from a padded 13-mer sequence, the context words of   ^^ A would be BCD, EFG, and HIJ.

### 2.11 Pre-training of the input modules

Pre-training was performed using a fastText-like technique to learn meaningful sub-word-level features and initialize the input layers. During pre-training, the model was trained to predict whether a pair of input words have a context relationship. To make the prediction, the embedding of each input word was first calculated as the sum of the embeddings of its sub-words. Then, the dot product of the embeddings of the two input words was computed and passed through a sigmoid activation. Binary cross-entropy was used as the loss function. The architecture of the input modules is provided in Supplementary Fig. S1.

### 2.12 Negative HLA binding data sampling

As mass spectrometry data contain only peptides that were bound to the HLA molecules, it is necessary to generate synthetic negative data points for training the model. A standard assumption is that a randomly generated peptide is unlikely to be able to bind to an HLA protein. Here, a random peptide was generated by first sampling the length from the distribution of HLA-bound peptides. Then, the amino acid for each position was uniformly sampled. Finally, each generated peptide was randomly paired with an HLA allele in the dataset. This whole process was repeatedly performed for each batch of data during training.

### 2.13 Prediction head

The prediction head outputs the probability of binding between an input peptide and an input HLA allele. The prediction head receives inputs from the peptide input module and the allele input modules and passes them to the classification head, as shown in Fig. 1. The input peptide length was increased from 9 during pre-training to 45 amino acids to accommodate longer peptides. Embeddings for unseen sub-words were initialized with a uniform distribution.

Word-level representations for the input peptide and HLA allele were each fed through a separate fully connected layer with 250 neurons and a dropout layer with probability of 0.5. The outputs were flattened, concatenated, and passed through two fully connected layers, each with 240 neurons, a dropout layer with probability of 0.4, and a fully connected layer with sigmoid activation that produces the prediction. Rectified linear unit activation was used in all other fully connected layers. SGD optimizer with a learning rate of 0.01 was used. The detailed architecture of the prediction head is provided in Supplementary Fig. S2.

### 2.14 Architecture and hyperparameter tuning

For the peptide and HLA allele input heads, fully connected layer, GRU layer, and LSTM layer were tested. The numbers of GRU and LSTM units were varied between 32, 64, 128, and 256. The GRU and LSTM layers were applied to either only the peptide input head or both the peptide and allele input heads. Employing GRU and LSTM did not yield superior performance over other pre-training configurations, which is likely due to the long input sequences (Chung *et al.* 2014) (372 amino acids for HLA allele). Hence, fully connected layers were selected for the input heads. The word size for skip-gram embedding was set at three amino acids based on a number of prior works (Phloyphisut *et al.* 2019, Khanal *et al.* 2020, He *et al.* 2021, Ibtehaz *et al.* 2023), and the fact that the input peptide lengths for this task are quite short.

For the embedding dimension, a grid search was conducted with dimensions of 16, 32, 64, 128, and 1024. Although using larger embedding dimensions tended to yield higher areas under the curve (AUC), the overall performance gain was negligible. For instance, increasing the dimension from 32 to 1024 only improved the AUC by 0.0034 but required significantly more memory. Consequently, the embedding dimension of 32 was selected.

During pre-training, a grid search was performed to identify an optimal distance threshold for defining the 3D structural context for allele embedding. Thresholds ranging from 5 to 65 Å, with a step size of 5 Å, were explored. The threshold of 45 Å was found to be optimal. Furthermore, a combined pre-training strategy where both the peptide and allele input heads were trained simultaneously was also evaluated. However, that technique only slightly improved the overall performance while hurting the performance on alleles with small amounts of training data.

## 3 Results

### 3.1 Performance comparison on mass spectrometry-based HLA binding data

**Figure 3.**
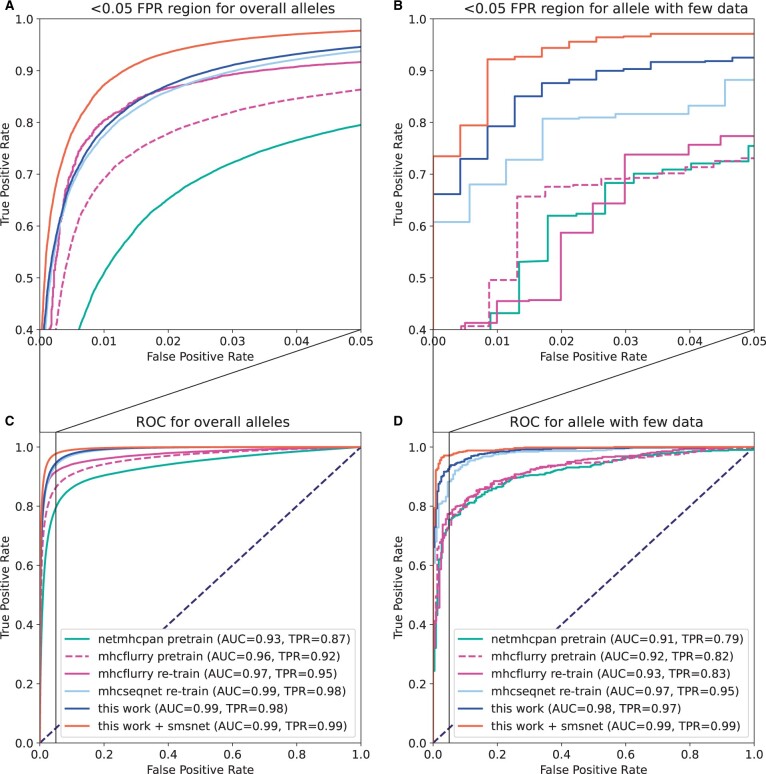
Performance comparisons with existing approaches on publicly available mass spectrometry data. The upper panels, (A) and (B), depict the zoomed-in ROC curves on the region with false positive rate ranging from 0.00 to 0.05. The lower panels, (C) and (D), show the full curves.

To provide a fair evaluation, only public HLA binding data from IEDB were used to evaluate all models. Additional peptide–HLA pairs obtained from the re-analysis of peptidomics datasets were included only for training because they have not been carefully validated. Furthermore, as most models can already achieve very high performance on HLA alleles with thousands of positive peptides, a separate evaluation considering only 45 HLA alleles, each with fewer than 200 known positive peptides, was also performed. In total, there are 2464 positive peptides associated with these 45 alleles. The ROC curves and performance summary are shown in Fig. 3 and Table 1. The precision–recall (PR) curves are shown in Supplementary Fig. S3.

**Table 1.** Performance comparison on IEDB mass spectrometry-based HLA binding data.

Method	AUC^a^	TPR^a^^,^^c^	AUC^b^	TPR^b^^,^^c^	AUPRC^a^	AUPRC^b^	*F*1^a^^,^^c^	*F*1^b^^,^^c^
NetMHCPan	0.9312	0.8681	0.9118	0.7901	0.9713	0.9619	0.9072	0.8627
MHCFlurry	0.9589	0.9171	0.9200	0.8199	0.9836	0.9698	0.9333	0.8803
MHCFlurry re-trained	0.9723	0.9476	0.9252	0.8260	0.9891	0.9685	0.9488	0.8834
MHCSeqNet re-trained	0.9868	0.9803	0.9748	0.9546	0.9943	0.9900	0.9649	0.9525
This work	**0.9884** ^d^	**0.9842**	**0.9842**	**0.9694**	**0.9950**	**0.9938**	**0.9668**	**0.9596**
This work + SMSNet data	**0.9936**	**0.9935**	**0.9930**	**0.9881**	**0.9972**	**0.9972**	**0.9712**	**0.9683**

a Evaluated on all alleles.

b Evaluated on 45 alleles amounts of training data (<200 positive peptides).

c True positive rates and *F*1 scores were measured at the 5% false discovery rate cutoff.

d Bolded values indicate the top two performance scores.

Our technique consistently outperforms prior works on both the whole dataset and especially on the set of 45 alleles with low amounts of training data. On the whole dataset, this work achieved the highest AUC of 0.9884 and the highest *F*1 score of 0.9668 at 5% false discovery rate. On the set of 45 alleles with low amounts of training data, this work achieved the highest AUC of 0.9842 and the highest *F*1 score of 0.9596. In contrast, NetMHCPan and MHCFlurry achieved AUCs of 0.9252 or lower and *F*1 scores of 0.8834 or lower on these alleles. Re-training MHCFlurry on our data split only slightly improved its performance (Table 1, MHCFlurry re-trained). More performance comparisons at 1% FDR threshold and on additional metrics are provided in Supplementary Tables S1 and S2.

### 3.2 Impact of additional training data

Incorporation of newly discovered peptide–HLA pairs from SMSNet improved prediction performances across all metrics (Table 1). The true positive rates increased from 0.9842 to 0.9930 overall and from 0.9694 to 0.9881 for HLA alleles with small amounts of training data. Similarity, the *F*1 scores increased from 0.9668 to 0.9712 overall and from 0.9596 to 0.9683 for HLA alleles with small numbers of training data. Additionally, an ablation analysis (Table 2) indicates that the magnitude of improvement due to addition of new data is almost as substantial as the improvement achieved through pre-training techniques. It should be noted that these additional peptide–HLA pairs were identified from the same mass spectrometry data of HLA peptidomics experiments (Abelin *et al.* 2017, Sarkizova *et al.* 2020) that have been analyzed and reported in public databases. Hence, these new data points should not affect the data distribution and only increases the training dataset size. More ablation results at 1% FDR threshold and on additional metrics are provided in Supplementary Tables S3 and S4.

**Table 2.** Ablation study on the impact of pre-training design choices.

Method	AUC^a^	TPR^a^^,^^c^	AUC^b^	TPR^b^^,^^c^
No pre-training	0.9852	0.9784	0.9634	0.9320
Peptide pre-training only	0.9882	0.9843	0.9829	0.9643
LSTM on peptide	0.9883	**0.9844**	0.9795	0.9626
LSTM on peptide + allele	0.9864	0.9812	0.9776	0.9643
GRU on peptide	0.9863	0.9819	0.9811	**0.9694**
GRU on peptide + allele	0.9867	0.9818	0.9775	0.9592
This work	**0.9884** ^d^	0.9842	**0.9842**	**0.9694**
This work + SMSNet data	**0.9936**	**0.9930**	**0.9935**	**0.9881**

a Evaluated on all alleles.

b Evaluated on 45 alleles with small number of training data (<200 positive peptides).

c True positive rates were measured at the 5% false discovery rate cutoff.

d Bolded values indicate the top two performance scores.

### 3.3 Transfer learning for binding affinity prediction

The potential usefulness of our approach was additionally evaluated on the HLA binding affinity (IC50) prediction task. Three configurations of our model were explored. First, the model was trained on binding affinity data from scratch without any pre-training. Second, the model was pre-trained on the HLA binding prediction task, without using new peptide–HLA pairs from SMSNet, before being fine-tuned on binding affinity data. Third, the model was pre-trained on the HLA binding prediction task, with additional data from SMSNet, before being fine-tuned on binding affinity data. Results in Table 3 show that our base architecture achieved a similar performance as MHCFlurry while pre-training further improved the performance by 0.012–0.015 on both AUC (calculated at the IC50 cutoff of 500 nM) and log mean squared error. The addition of SMSNet data had almost no impact on the performance for this task. It should be noted that NetMHCPan is expected to achieve the highest performance because the majority of the test set may have been seen by the model.

**Table 3.** Performance comparison on binding affinity prediction.

Method	AUC^a^	Log MSE
MHCFlurry	0.9190	0.0329
This work w/o pre-training	0.9169	0.0314
This work	0.9316	0.0275
This work + SMSNet data	0.9325	0.0272
NetMHCPan	0.9553^b^	

a Evaluated at binding affinity (IC50) cutoff of 500 nM.

b NetMHCPan likely has seen these binding affinity data. The performance number was included for reference.

### 3.4 Evaluation on T cell epitopes

**Figure 4.**
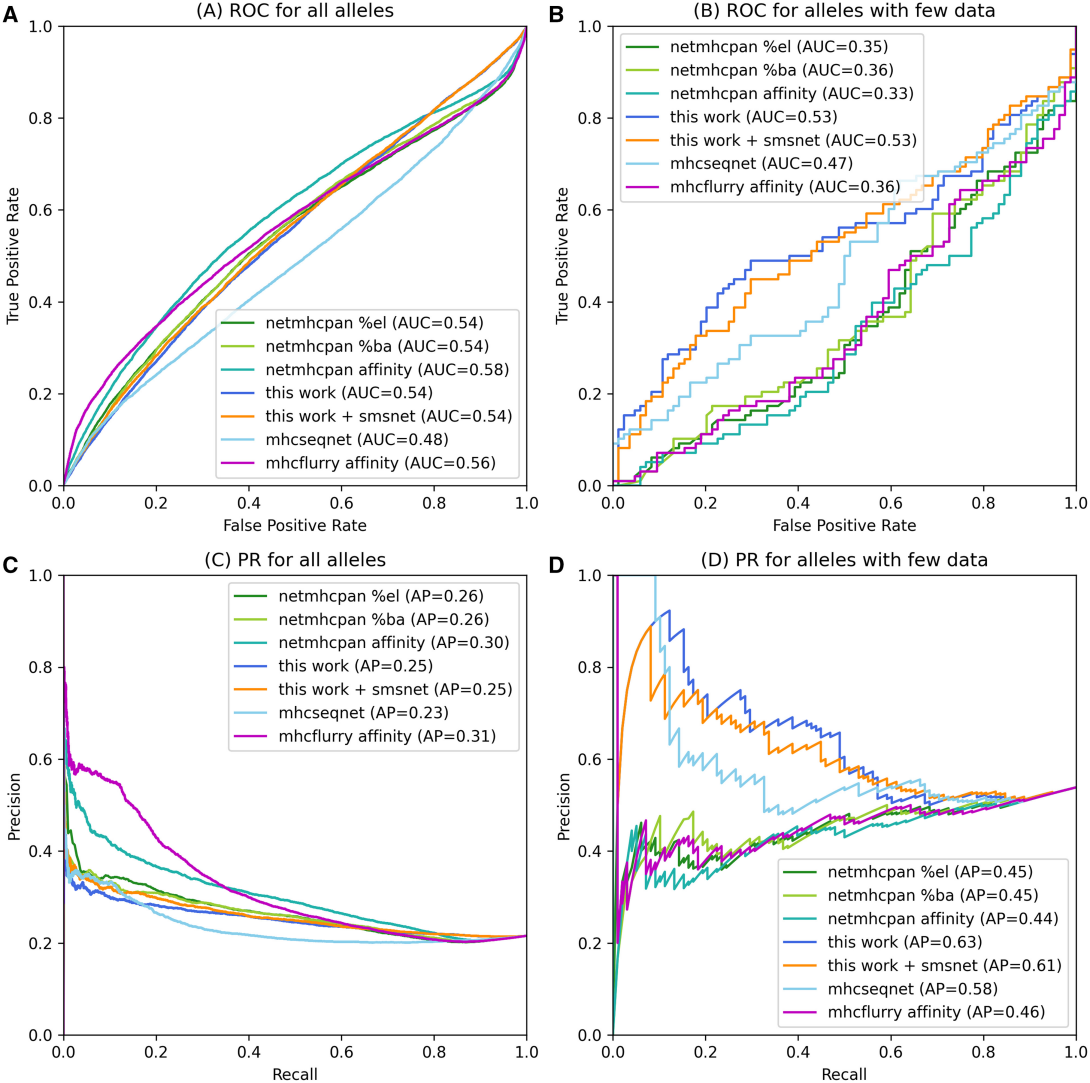
Performance evaluation on T cell epitopes from IEDB. The upper panels, (A) and (B), depict the ROC curves for all alleles and for alleles with low amounts of training data (<200 peptides), respectively. The lower panels, (C) and (D), show the corresponding PR curves. For NetMHCPan, predicted % rank of eluted ligand (%el), % rank of binding affinity (%ba), and raw affinity values were evaluated. For MHCSeqNet v1 and v2, predicted binding probabilities were evaluated. For MHCFlurry, predicted raw affinity values were evaluated. There were 184 peptide–HLA pairs (99 positive and 85 negative) across 35 HLA alleles with low amount of training data.

To illustrate the ability of MHCSeqNet2 to aid the screening of potential T cell epitopes, predicted HLA binding probabilities from MHCSeqNet were compared against MHCFlurry’s and NetMHCPan’s predictions (% rank of eluted ligand, %rank of binding affinity, and raw affinity) on known T cell epitopes from IEDB. Overall, MHCFlurry’s and NetMHCPan’s predicted raw affinity (IC50) values were slightly better than other scores for distinguishing T cell epitopes, with AUC of 0.56–0.58 compared to 0.54 (Fig. 4A). However, when restricting the evaluation to HLA alleles with low amount of training data, which is where MHCSeqNet2 was designed to address, both versions of MHCSeqNet2 maintain the same performance levels while NetMHCPan’s AUCs dropped to 0.33–0.36 (Fig. 4B). Similar behaviors were observed when examining the PR curves (Fig. 4C and D), with both versions of MHCSetNet2 achieving average precisions of 0.61–0.63 on HLA alleles with low amount of training data.

### 3.5 Learned allele embeddings match with binding motifs

**Figure 5.**
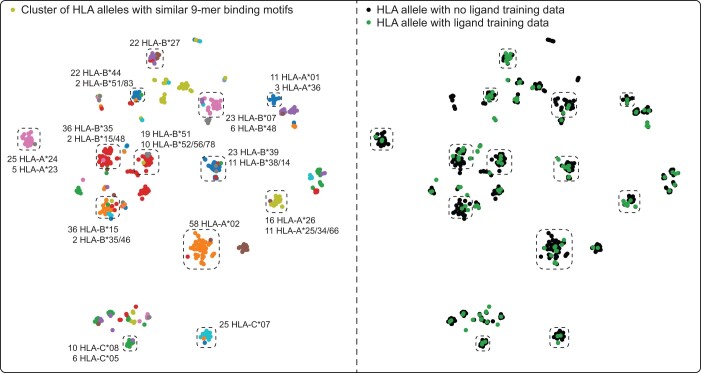
A region of the 2D t-SNE scatter plot of HLA allele embeddings. Coloring on the left panel is based on the DBSCAN clustering of known 9-mer binding motifs (in the form of position-specific amino acid frequency matrices), while the coloring on the right panel denote the absence/presence of ligand training data for each allele. The most frequent HLA alleles in each region of the plot are indicated.

To investigate whether our approach produces meaningful embeddings of HLA alleles, the embeddings were visualized using t-SNE with colors indicating HLA alleles with similar 9-mer binding motifs (DBSCAN of flattened position-specific amino acid frequency matrices) (Rapin *et al.* 2008). Overall, there was a good concordance between the learned embeddings and ligand specificities (Fig. 5, left panel, adjusted mutual information =0.60). Alleles with similar binding motifs received similar embeddings. Most importantly, our embedding can group alleles with and without ligand training data together (Fig. 5, right panel, black dots surrounding green dots). This strongly suggests that the model should be able to transfer knowledge from alleles with large amounts of training data to closely related alleles that lack training data.

### 3.6 Performance on peptides dissimilar to the training set

Lastly, because peptides with similar amino acid sequences tend to have similar binding affinity with an HLA allele, if the dataset was not carefully split to ensure that highly similar peptides were not spread across the training, validation, and test sets, there is a high risk of overfitting and obtaining inflated performance scores. Here, as the overall performances were above 0.99 in many metrics, an in-depth error analysis is needed to rule out the possibility of overfitting. To address this concern, the performance of the model was evaluated on multiple groups of peptides in the test set, which were split according to their minimal edit distance to peptides in the training set. The result indicates that the model performed equally well on all edit distance groups (Table 4), with no apparent bias toward peptides that are similar to those in the training set and no drop in performance on unseen peptides. Theses evidences suggest that the model did not overfit.

**Table 4.** Model performances on test peptides with various degrees of similarity to peptides in the training set.

Edit distance	This work	This work + SMSnet data
≤2	0.9592 (0.0068)^a^	0.9755 (0.0045)
3	0.9774 (0.0003)	0.9868 (0.0038)
4	0.9814 (0.0006)	0.9894 (0.0031)
≥5	0.9774 (0.0015)	0.9877 (0.0037)

a The average AUC and SD were reported.

### 3.7 Resource utilization and inference speed

The consumption of computing resources during inference was measured on a desktop equipped with an AMD Ryzen 9 5950x CPU (16 cores at 3.40 GHz), 64 GB of memory, and an NVIDIA GeForce RTX3090 GPU. The input batch size was set at 256. With the GPU, the model can make 15 931 predictions per second. Peak GPU memory usage was at 1057 MB. Without the GPU (CPU-only), 5938 predictions could be made per second. Furthermore, if the input data have been preloaded into the memory, the inference throughput can reach 68 627 and 16 993 predictions per second, with and without GPU, respectively.

## 4 Discussion

In this study, MHCSeqNet framework was further improved by incorporating a fastText-like technique to learn embeddings for the input peptides and HLA alleles and by utilizing additional mass spectrometry-based training data. This new version of MHCSeqNet achieved the highest performances across major metrics, including AUC, TPR, AUPRC, and *F*1, compared to other state-of-the-art models (Table 1 and Fig. 3). The performance boosts are especially pronounced on the set of 45 alleles with low amounts of training data (<200 positive peptides each), which is our primary objective. Ablation analysis indicated that both pre-training and additional data equally contributed to the improvement (Table 2). The learned embeddings for HLA alleles also matched well to known binding motifs (Fig. 5). Furthermore, our approach can be transferred to improve the prediction of HLA binding affinity as well (Table 3). These findings illustrate the strong power of pre-training, which not only improved the overall prediction performance, but also helped the model transfer knowledge from HLA alleles with large amounts of training data to related HLA alleles.

The architecture of MHCSeqNet differs from NetMHCPan and MHCFlurry in two key aspects, the input embedding strategy and the non-ensemble approach. MHCSeqNet incorporates dedicated amino acid sequence embedding layers while a static BLOSUM-based encoding (Nielsen *et al.* 2003) was used alongside extra information about the peptide, such as length and flanking sequences, in other studies. Our design choice facilitated the learning of sub-word features and seamlessly integrated sequence and structural contextual information during pre-training. The embeddings could also adapt to new data as the model is being trained on peptide–HLA pairs for the main prediction tasks. These characteristics likely explain why MHCSeqNet can generalize to diverse HLA alleles without requiring an ensemble of predictors like in other works.

Although the impact may be small, pre-training clearly improved the performance of the models on both binding probability and binding affinity prediction tasks, particularly on HLA alleles with small amounts of training data. It is interesting to note that the allele embedding module can be pre-trained almost as effectively using generic 9-mer peptides as using HLA-specific 3D structural context information (Table 2, “Peptide pre-training only” compared to “This work”). This may be because the 3D structural context information also involves parts of the HLA protein that do not contribute to the binding with antigen. Another possibility is that HLA structures provide only a limited understanding about the broad protein 3D structural context. Combining structural context data from multiple protein families for pre-training the allele embedding module may be a better approach.

Lastly, it is curious to note that the use of GRU and LSTM layers not only did not improve the overall performance but also reduced the generalization ability of the model on HLA alleles with low amount of training data (Table 2). This may resulted from a combination of overfitting and these layers’ limited ability to handle long HLA allele inputs (Chung *et al.* 2014), which are 372 amino acids in length.

## Supplementary Material

btad780_Supplementary_Data

## Data Availability

All data used to train and validate the model were obtained from public databases and publications, as listed in Section 2.1-2.6. Other resources required to run the model are provided in GitHub at https://github.com/cmb-chula/MHCSeqNet2.

## References

[btad780-B1] Abelin JG , Harjanto D, Malloy M et al Defining HLA-II ligand processing and binding rules with mass spectrometry enhances cancer epitope prediction. Immunity 2019;51:766–79.e17. 31495665 10.1016/j.immuni.2019.08.012

[btad780-B2] Abelin JG , Keskin DB, Sarkizova S et al Mass spectrometry profiling of HLA-associated peptidomes in mono-allelic cells enables more accurate epitope prediction. Immunity 2017;46:315–26. 28228285 10.1016/j.immuni.2017.02.007 PMC5405381

[btad780-B3] Andreatta M , Nielsen M. Gapped sequence alignment using artificial neural networks: application to the MHC class I system. Bioinformatics 2015;32:511–7. 26515819 10.1093/bioinformatics/btv639 PMC6402319

[btad780-B4] Bojanowski P , Grave E, Joulin A et al Enriching word vectors with subword information. TACL 2017;5:135–46.

[btad780-B5] Chen B , Khodadoust MS, Olsson N et al Predicting HLA class II antigen presentation through integrated deep learning. Nat Biotechnol 2019;37:1332–43. 31611695 10.1038/s41587-019-0280-2 PMC7075463

[btad780-B6] Chung J , Gulcehre C, Cho K et al Empirical Evaluation of Gated Recurrent Neural Networks on Sequence Modeling. In: *NIPS 2014 Workshop on Deep Learning *2014.

[btad780-B7] Edgar RC. MUSCLE: a multiple sequence alignment method with reduced time and space complexity. BMC Bioinformatics 2004;5:113. 15318951 10.1186/1471-2105-5-113 PMC517706

[btad780-B8] He W , Wang Y, Cui L et al Learning embedding features based on multisense-scaled attention architecture to improve the predictive performance of anticancer peptides. Bioinformatics 2021;37:4684–93. 34323948 10.1093/bioinformatics/btab560

[btad780-B9] Ibtehaz N , Sourav SMSH, Bayzid MS et al Align-gram: rethinking the skip-gram model for protein sequence analysis. Protein J 2023;42:135–46. 36977849 10.1007/s10930-023-10096-7

[btad780-B10] Karunratanakul K , Tang H-Y, Speicher DW et al Uncovering thousands of new peptides with sequence-mask-search hybrid de novo peptide sequencing framework. Mol Cell Proteomics 2019;18:2478–91. 31591261 10.1074/mcp.TIR119.001656 PMC6885704

[btad780-B11] Keşmir C , Nussbaum AK, Schild H et al Prediction of proteasome cleavage motifs by neural networks. Protein Eng 2002;15:287–96. 11983929 10.1093/protein/15.4.287

[btad780-B12] Khanal J , Tayara H, Chong KT. Identifying enhancers and their strength by the integration of word embedding and convolution neural network. IEEE Access 2020;8:58369–76.

[btad780-B13] Kruger S , Ilmer M, Kobold S et al Advances in cancer immunotherapy 2019 – latest trends. J Exp Clin Cancer Res 2019;38:268. 31217020 10.1186/s13046-019-1266-0 PMC6585101

[btad780-B14] Marco MD , Schuster H, Backert L et al Unveiling the peptide motifs of HLA-C and HLA-G from naturally presented peptides and generation of binding prediction matrices. J Immunol 2017;199:2639–51. 28904123 10.4049/jimmunol.1700938

[btad780-B15] McCarthy EF. The toxins of William B. Coley and the treatment of bone and soft-tissue sarcomas. Iowa Orthop J 2006;26:154–8. 16789469 PMC1888599

[btad780-B16] Menezes Teles e Oliveira D , Melo Santos de Serpa BrandÃ£o R, Claudio Demes da Mata Sousa L et al pHLA3D: an online database of predicted three-dimensional structures of HLA molecules. Hum Immunol 2019;80:834–41. 31239187 10.1016/j.humimm.2019.06.009

[btad780-B17] Nielsen M , Lundegaard C, Worning P et al Reliable prediction of T-cell epitopes using neural networks with novel sequence representations. Protein Sci 2003;12:1007–17. 12717023 10.1110/ps.0239403 PMC2323871

[btad780-B18] O'Donnell TJ , Rubinsteyn A, Laserson U. MHCflurry 2.0: improved pan-allele prediction of MHC Class I-presented peptides by incorporating antigen processing. Cell Syst 2020;11:42–8.e7. 32711842 10.1016/j.cels.2020.06.010

[btad780-B19] Phloyphisut P , Pornputtapong N, Sriswasdi S et al MHCSeqNet: a deep neural network model for universal MHC binding prediction. BMC Bioinformatics 2019;20:270. 31138107 10.1186/s12859-019-2892-4 PMC6540523

[btad780-B20] Rapin N , Hoof I, Lund O et al MHC motif viewer. Immunogenetics 2008;60:759–65. 18766337 10.1007/s00251-008-0330-2 PMC2613509

[btad780-B21] Reynisson B , Alvarez B, Paul S et al NetMHCpan-4.1 and NetMHCIIpan-4.0: improved predictions of MHC antigen presentation by concurrent motif deconvolution and integration of MS MHC eluted ligand data. Nucleic Acids Res 2020;48:W449–54. 32406916 10.1093/nar/gkaa379 PMC7319546

[btad780-B22] Sarkizova S , Klaeger S, Le PM et al A large peptidome dataset improves HLA class I epitope prediction across most of the human population. Nat Biotechnol 2020;38:199–209. 31844290 10.1038/s41587-019-0322-9 PMC7008090

[btad780-B23] Solleder M , Guillaume P, Racle J et al Mass spectrometry based immunopeptidomics leads to robust predictions of phosphorylated HLA class I ligands. Mol Cell Proteomics 2019;19:390–404. 31848261 10.1074/mcp.TIR119.001641 PMC7000122

[btad780-B24] Sricharoensuk C , Boonchalermvichien T, Muanwien P et al Unsupervised mining of HLA-I peptidomes reveals new binding motifs and potential false positives in the community database. Front Immunol 2022;13:847756. 35386688 10.3389/fimmu.2022.847756 PMC8977642

[btad780-B25] Teles e Oliveira DM , Marroquim MSC, de Serpa BrandÃ£o RMS et al pHLA3D: updating the database of predicted three-dimensional structures of HLA with HLA-DR, HLA-DQ and HLA-DP molecules. Hum Immunol 2021;82:8–10. 33129577 10.1016/j.humimm.2020.10.007

[btad780-B26] The UniProt Consortium. UniProt: the universal protein knowledgebase in 2021. Nucleic Acids Res 2020;49:D480–9. 10.1093/nar/gkaa1100 PMC7778908 33237286

[btad780-B27] Vita R , Mahajan S, Overton JA et al The Immune Epitope Database (IEDB): 2018 update. Nucleic Acids Res 2018;47:D339–43. 10.1093/nar/gky1006 PMC6324067 30357391

[btad780-B28] Wells DK , van Buuren MM, Dang KK. Key parameters of tumor epitope immunogenicity revealed through a consortium approach improve neoantigen prediction. Cell 2020;183:818–34.e13. 33038342 10.1016/j.cell.2020.09.015 PMC7652061

[btad780-B29] Wieczorek M , Abualrous ET, Sticht J et al Major histocompatibility complex (MHC) class I and MHC class II proteins: conformational plasticity in antigen presentation. Front Immunol 2017;8:292. 28367149 10.3389/fimmu.2017.00292 PMC5355494

[btad780-B30] Xie X , Han Y, Zhang K. MHCherryPan. a novel model to predict the binding affinity of pan-specific class I HLA-peptide. International Journal of Data Mining and Bioinformatics 2020;24:201–19.

