# Supplementary material for: MHCSeqNet2—improved peptide-class I MHC binding prediction for alleles with low data
Source: Bioinformatics. 2023 Dec 28;40(1):btad780. doi: 10.1093/bioinformatics/btad780 (PMC10783953; doi:10.1093/bioinformatics/btad780)
Supplement: btad780_Supplementary_Data [file btad780_supplementary_data.pdf]

1. SUPPLEMENTARY TABLES

Table S1. HLA binding prediction performance comparison at 1% false discovery rate (FDR) threshold

Name	FDR=0.01							
	Accuracy		F1		PPV		TPR	
	Overall	Low data	Overall	Low data	Overall	Low data	Overall	Low data
NetMHCPan	0.6974	0.5761	0.7279	0.5617	0.9900	0.9900	0.5756	0.3921
MHCFlurry	0.2860	0.6441	0.0008	0.6727	0.9900	0.9900	0.0004	0.5094
MHCFlurry re-trained	0.2899	0.5855	0.0124	0.5849	0.9900	0.9900	0.0062	0.4151
MHCSeqNet re-trained	0.9004	0.8006	0.9258	0.8392	0.9900	0.9907	0.8694	0.7279
This work	0.9102	0.9053	0.9336	0.9296	0.9900	0.9904	0.8832	0.8759
This work + SMSNet data	0.9548	0.9672	0.9676	0.9767	0.9900	0.9895	0.9463	0.9643

Table S2. HLA binding prediction performance comparison at 5% false discovery rate (FDR) threshold

Name	FDR=0.05							
	Accuracy		F1		PPV		TPR	
	Overall	Low data	Overall	Low data	Overall	Low data	Overall	Low data
NetMHCPan	0.8751	0.8258	0.9072	0.8627	0.9500	0.9500	0.8681	0.7901
MHCFlurry	0.9063	0.8399	0.9333	0.8803	0.9500	0.9503	0.9171	0.8199
MHCFlurry re-trained	0.9270	0.8466	0.9488	0.8834	0.9500	0.9494	0.9476	0.8260
MHCSeqNet re-trained	0.9490	0.9319	0.9649	0.9525	0.9500	0.9503	0.9803	0.9546
This work	0.9517	0.9417	0.9668	0.9596	0.9500	0.9500	0.9842	0.9694
This work + SMSNet data	0.9580	0.9539	0.9712	0.9683	0.9500	0.9493	0.9935	0.9881

Table S3. Impact of pre-training on HLA binding prediction at 1% false discovery rate (FDR) threshold

Name	FDR=0.01							
	Accuracy		F1		PPV		TPR	
	Overall	Low data	Overall	Low data	Overall	Low data	Overall	Low data
No pre-training	0.8844	0.7306	0.9128	0.7692	0.9900	0.9893	0.8467	0.6293
Pre-train with only 9-mers	0.9078	0.8811	0.9316	0.9099	0.9900	0.9900	0.8798	0.8418
This work with GRU layer	0.8941	0.8471	0.9207	0.8811	0.9900	0.9894	0.8605	0.7942
This work	0.9102	0.9053	0.9336	0.9296	0.9900	0.9904	0.8832	0.8759

Table S4. Impact of pre-training on HLA binding prediction at 5% false discovery rate (FDR) threshold

Name	FDR=0.05							
	Accuracy		F1		PPV		TPR	
	Overall	Low data	Overall	Low data	Overall	Low data	Overall	Low data
No pre-training	0.9478	0.9163	0.9640	0.9408	0.9500	0.9497	0.9784	0.9320
Pre-train with only 9-mers	0.9518	0.9381	0.9669	0.9570	0.9500	0.9497	0.9843	0.9643
This work with GRU layer	0.9502	0.9417	0.9657	0.9596	0.9500	0.9500	0.9819	0.9694
This work	0.9517	0.9417	0.9668	0.9596	0.9500	0.9500	0.9842	0.9694

2. SUPPLEMENTARY FIGURES

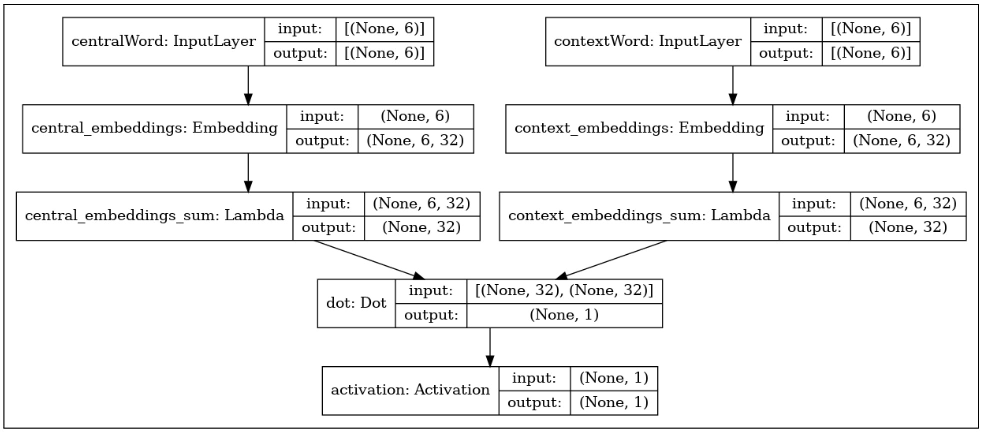

Fig. S1. Architecture of the peptide and allele input modules during pre-traineding stage. The dimension of each layer is indicated.

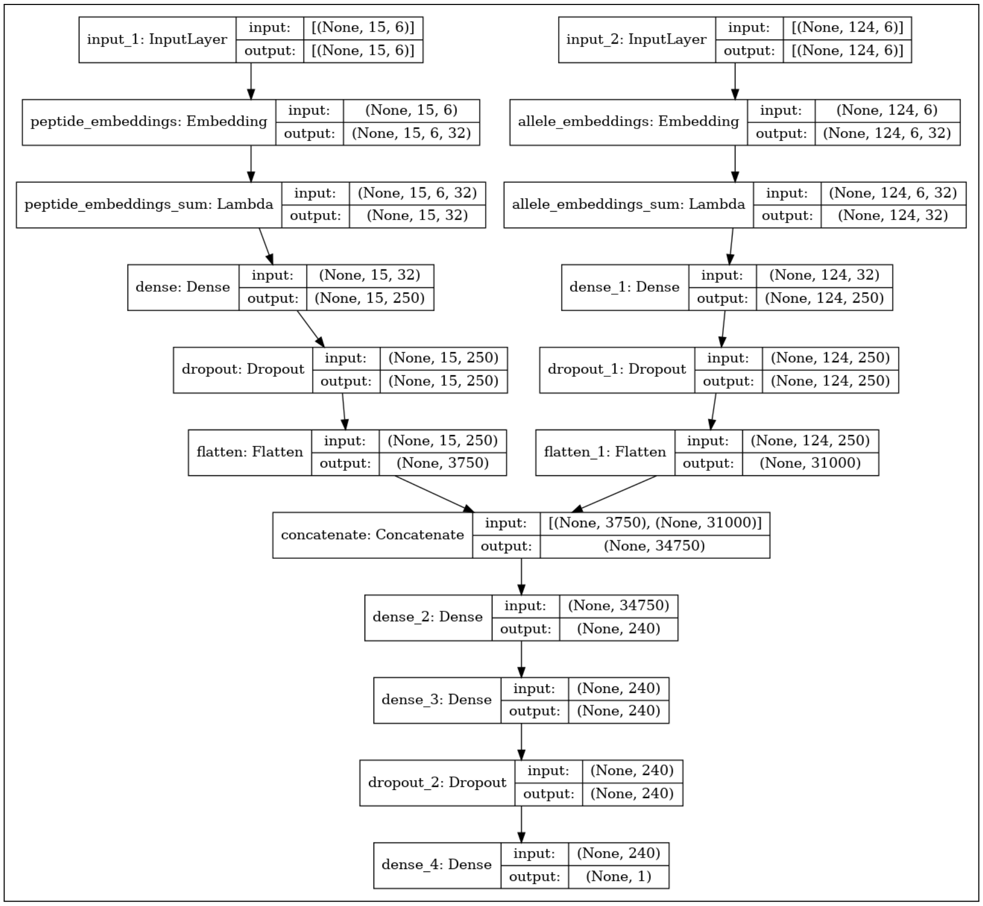

Fig. S2. Architecture of the full model during training of the main task. The dimension of each layer is indicated.

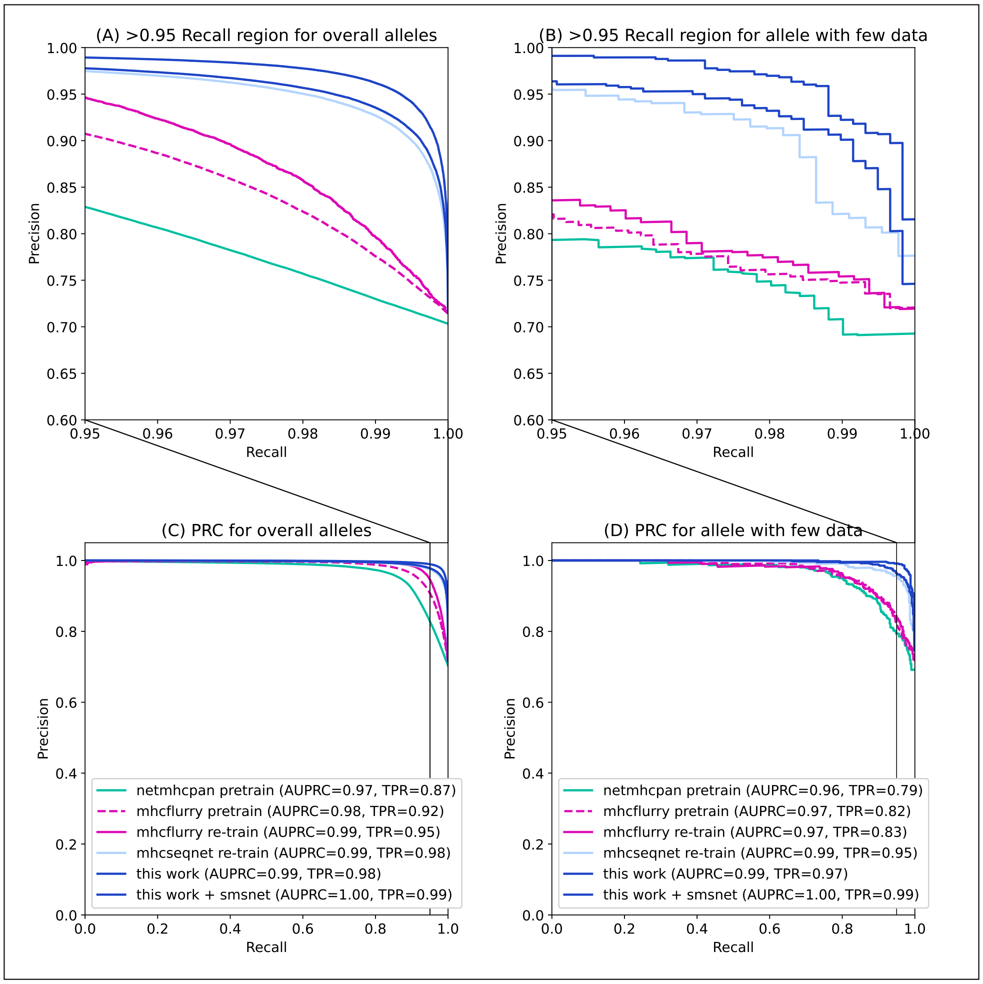

Fig. S3. Performance comparisons with existing approaches on publicly available mass spectrometry data. The upper panels, (A) and (B), depict the zoomed-in precision-recall curves on the region with high recall ranging from 0.95 to 1.00. The lower panels, (C) and (D), show the full curves.
